# Association between serum manganese and serum klotho in a 40–80-year-old American population from NHANES 2011–2016

**DOI:** 10.3389/fragi.2023.1120823

**Published:** 2023-03-08

**Authors:** Guoyu Guan, Jiasheng Cai, Songbai Zheng, Yanzhen Xiang, Shijin Xia, Yixuan Zhang, Jiaqiang Shi, Jun Wang

**Affiliations:** ^1^ Department of Geriatrics, Huadong Hospital, Shanghai Medical College Fudan University, Shanghai, China; ^2^ Department of Cardiology, Huadong Hospital, Shanghai Medical College Fudan University, Shanghai, China; ^3^ Department of General Practice, Huadong Hospital, Shanghai Medical College Fudan University, Shanghai, China

**Keywords:** manganese, klotho, lifespan, NHANES, biomarker of anti-aging, longevity, nutrition

## Abstract

**Objectives:** Manganese is one of the essential trace elements that are required by the human body. Klotho protein is a classic anti-aging marker. The association between the levels of serum manganese and serum klotho in individuals between the ages of 40–80 in the United States remains unclear.

**Methods:** Data for this cross-sectional study was obtained from the National Health and Nutrition Examination Survey (NHANES 2011–2016) in the United States. We performed multiple linear regression analyses to investigate the association between the levels of serum manganese and serum klotho. Furthermore, we performed a fitted smoothing curve according to a restricted cubic spline (RCS). Stratification and subgroup analyses were performed for further verification of the results.

**Results**: Weighted multivariate linear regression analysis showed that serum manganese levels were independently and positively associated with serum klotho levels (*β* = 6.30, 95% confidence interval: 3.30–9.40). Kruskal–Wallis test showed that participants with higher manganese quartiles had higher serum klotho levels (Q1: 808.54 ± 256.39 pg/mL; Q2: 854.56 ± 266.13 pg/mL; Q3: 865.13 ± 300.60 pg/mL; and Q4: 871.72 ± 338.85 pg/mL, *p* < 0.001). The RCS curve indicated that the association between the levels of serum manganese and serum klotho was non-linear. Furthermore, a significantly positive association was found between serum manganese and serum klotho levels in the majority of subgroups.

**Conclusion:** A non-linear and positive association was found between the levels of serum manganese and serum klotho in individuals aged 40–80 in the United States according to the NHANES (2011–2016).

## 1 Introduction

Aging is involved in the pathological alterations of nearly all tissues or organs of the body, leading eventually to debilitating or chronic diseases (Prud’homme et al., 2022). In the past 30 years, klotho has been one of the classic anti-aging biomarkers. This protein performs a considerable role in regulating the activity of fibroblast growth factor and maintaining phosphate equilibrium in the body (Kuro-o et al., 1997; Buchanan et al., 2020). The core members of the klotho family proteins are *a*-klotho, *ß*-klotho, and γ-klotho (Hu et al., 2013; Kuro and Moe, 2017; Kuro, 2019), which are isomers of each other and are single-pass transmembrane proteins. Soluble *a*-klotho is found in the cerebrospinal fluid, blood, and urine (Imura et al., 2004; Kurosu et al., 2005; Akimoto et al., 2012), which is hereby referred to as “klotho” in this study. *In vivo* experiments and clinical studies show that low serum klotho levels accelerate senescence (Xiao et al., 2004; Dërmaku-Sopjani et al., 2013) and death (Kresovich and Bulka, 2022), and are also associated with an increased risk of age-related diseases such as atherosclerosis (Pan et al., 2018; Chen et al., 2021), chronic kidney disease (Manya et al., 2010; Drew et al., 2017), type 2 diabetes mellitus (T2DM) (Nie et al., 2017), metabolic syndrome (Kim et al., 2019), and pulmonary emphysema (Suga et al., 2000). A previous study showed that klotho increased resistance to oxidative stress by upregulating superoxide dismutase (SOD) (Kurosu et al*.*, 2005) and slowed aging by inhibiting insulin and insulin-like growth factor-1 (IGF-1) signaling, which was affected by nutritional status (Partridge and Gems, 2002).

Manganese is an essential micronutrient without adequate levels in virtually all types of diets (Parmalee and Aschner, 2016). Previous studies showed that manganese was involved in many crucial physiological activities of cells (Malecki et al., 1994; Aschner and Aschner, 2005; Aschner and Erikson, 2017) such as regulating immune functions, stabilizing blood sugar levels, maintaining cellular energy, and resisting oxidative stress. Previous clinical trials have shown that low serum manganese levels were associated with a higher risk of hypertension, renal dysfunction, T2DM, and impaired longevity (Koh et al., 2014; Lv et al., 2021; Zhang et al., 2022). The increase in antioxidant levels is closely related to the longevity of the body (Finkel and Holbrook, 2000). Manganese can regulate the expression and activity of manganese superoxide dismutase (MnSOD) (Smith et al., 2017; Li and Yang, 2018) and then decrease the oxidative stress of the body, to slow down aging (Malecki et al., 1994).

Based on the circumstantial evidence that both serum klotho and serum manganese levels decrease with older age (Oulhote et al., 2014) and can decrease oxidative stress by regulating the activity of SOD to promote longevity (Kurosu et al*.*, 2005; Malecki et al., 1994). Furthermore, nutritional intake can affect insulin and IGF-1 signaling, which is related to the anti-aging properties of klotho (Partridge and Gems, 2002). Therefore, we reasonably speculated that serum manganese, which is an essential micronutrient, might be associated with serum klotho. If this correlation is confirmed through statistical and pathophysiological analyses, we propose that serum manganese levels can be a potential biomarker of klotho. As a proper index, serum manganese levels might rightly reflect malnutrition in the process of aging. Therefore, a large-scale cross-sectional study was performed to investigate the association between serum manganese and serum klotho levels in individuals between the ages of 40–80 in the United States according to the NHANES (2011–2016).

## 2 Materials and methods

### 2.1 Demographics of the study participants

**FIGURE 1:**
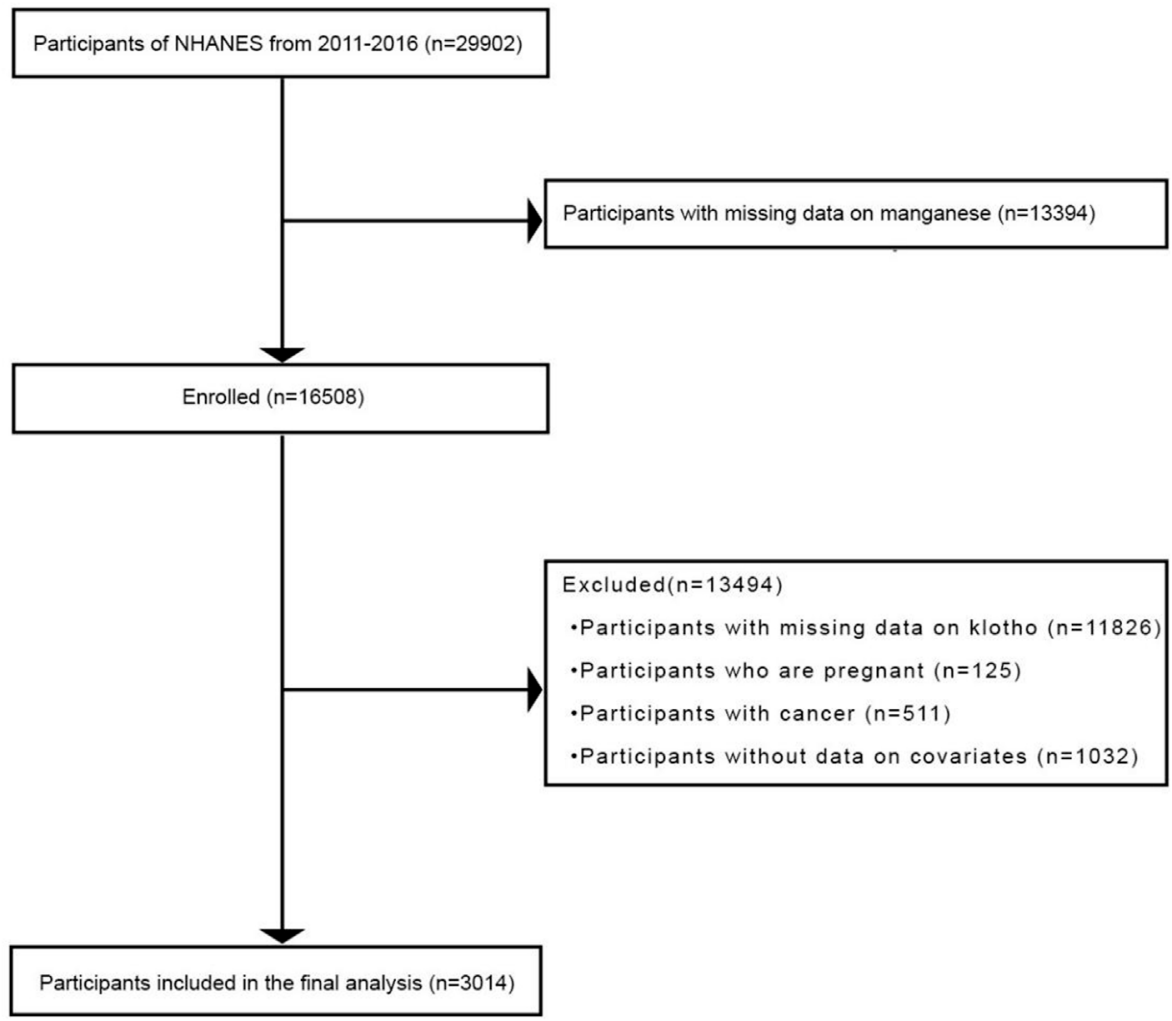
Flowchart depicting the process of participant recruitment in this study.

The National Health and Nutrition Examination Survey (NHANES) is an accessible database from the United States that contains questionnaire data on national health and nutritional status (Yang et al., 2014) as well as the results of laboratory and imaging tests (Xiao et al., 2021). Continuous information on the non-institutionalized US population was included in the NHANES, with every 2 years representing 1 cycle. This retrospective study analyzed health information collected from 29,902 subjects from NHANES during 2011–2016 (2011–2012, 2013–2014, and 2015–2016). Information about serum manganese and serum klotho was completely provided only in these years. This survey was conducted periodically under the approval of the Institutional Review Board (ERB) of the National Center for Health Statistics (NCHS), and each individual provided signed informed consent (Yang et al., 2014). The participants who lacked information on manganese (n = 13,394) or klotho (n = 11,826) were excluded. Pregnant women (n = 125) and cancer patients (n = 511) were also excluded from the study. In addition, subjects without covariates (n = 1032) were removed, which included educational attainment (n = 120), income-to-poverty ratio (PIR; n = 329), alcohol use (n = 285), body mass index (BMI, n = 27), diabetes (n = 119), 24-h total energy intake (n = 151), and smoking habit (n = 1). Eventually, we enrolled a total of 3014 participants in this study. A flowchart depicting the subject screening process is shown in Figure 1.

### 2.2 Manganese measurements

The whole blood of participants was collected and dispatched to the Centers for Disease Control and Prevention, Division of Laboratory Sciences, and National Center for Environmental Health (Atlanta, GA, United States) for analyses. The blood samples were stored at 30°C and then diluted (1 part sample +48 parts diluent1 + part water). Inductively coupled plasma-mass spectrometer (ICP-MS) with dynamic reaction cell technology) separates Mn under oxygen pressurization. For internal quality control, spiked pools was used, and external calibration utilized standard reference materials to meet the quality control standard (Gbemavo and Bouchard, 2021). After the detection, the mass spectrometer was cleaned in an aqueous solution of 0.01% ammonium pyrrolidinedithiocarbamate (APDC) for the next usage.

### 2.3 Klotho measurements

Northwest Lipid Metabolism and Diabetes Research Laboratories affiliated with the University of Washington used an ELISA kit (Fujioka Immunobiology Laboratory, Japan) for detecting the serum klotho levels of the whole blood samples (Alkalbani et al., 2022). All samples were stored under appropriate freezing (−80°C) conditions before conducting the assay. Two parallel holes were created in the ELISA plate to measure the klotho concentration of quality control samples, and the average value was considered as the final concentration. The serum klotho levels among healthy people fluctuated from 285.8 to 1638.6 pg/mL. The actual minimum measured concentration of this ELISA kit was 4.33 pg/mL, which is higher than the low limit value reported by the producer (6.15 pg/mL) (Cai et al., 2023). In addition, all procedures were conducted under laboratory-specified criteria.

### 2.4 Covariate information

We explored the association between serum manganese and serum klotho levels after adjusting for covariates selected in accordance with the literature (Xiao et al., 2021; Kim et al., 2022; Kresovich and Bulka, 2022). Sociodemographic characteristics were obtained using computer-assisted questionnaires, which included age, gender, race, educational attainment, marital status, and PIR. The health-related characteristics were also considered, which included the smoking habit (people who have or have never smoked >100 cigarettes in their lifetime), alcohol use (people who have or have never drunk >12 alcoholic beverages in a year), physical activity, BMI, and the 24-h total energy intake. In addition, two variables (i.e., the presence of diabetes and hypertension) were considered as medical comorbidities based on the response to the following question: “Has a doctor or other health professional ever told you that you suffered from diabetes/hypertension?” Most studies focusing on the two variables of blood manganese and blood klotho collected information on these two diseases (Wang et al., 2016; Nie et al., 2017; Gbemavo and Bouchard, 2021; Alkalbani et al., 2022; Chen et al., 2022; Cai et al., 2023).

### 2.5 Statistical analyses

In this investigation, the data on normal distribution were displayed as the mean ± standard deviation (SD), while the data on the skewed distribution were presented as the median (interquartile range: IQR). The categorical variables were demonstrated as a percentage (%). A weighted multiple linear regression analysis was performed to estimate the association of the serum manganese levels with the serum klotho concentrations in three different models. In Model 1, there was no adjustment for any variables. In Model 2, there was an adjustment for only 3 variables, that is, age, gender, and race. Building on Model 2, in Model 3, there was a further adjustment for the following variables: educational attainment, marital status, PIR, smoking habit, alcohol use, physical activity, BMI, 24-h total energy intake, diabetes, and hypertension. To illustrate the stability of the present results, the association between the serum manganese and serum klotho level was obtained with due consideration of the manganese concentration as a continuous variable and a categorical variable, respectively. We also transformed the raw data of these two variables by the lg function and then performed a fitted smoothing curve of the correlation between the serum manganese and serum klotho levels based on the restricted cubic spline (RCS). To determine the threshold, the non-segmented and segmented regression models were compared by the log-likelihood ratio test. Moreover, stratified and subgroup analyses were conducted considering age, gender, race, diabetes, and hypertension as stratified variables, respectively. The statistical analysis software used in this study included EmpowerStats and R version 4.2.0. Two-sided *p* < 0.05 was considered to indicate statistical significance.

## 3 Results

### 3.1 The baseline characteristics of the participants

In this study, a total of 3014 participants were included from the NHANES (2011–2016) in the United States. The specific screening of the participants is shown in Figure 1. According to the measured distribution of serum manganese levels (Q1: ≤7.27 μg/L; Q2: 7.27–9.10 μg/L; Q3: 9.10–11.55 μg/L; and Q4: >11.55 μg/L), participants were divided into quartiles based on the intuitively describing weighted demographic and medical characteristics (Table 1). Overall, the average age of participants was 56.83 ± 10.50, 50.13% were female, 38.79% belonged to the non-Hispanic white race, 55.01% had educational qualifications beyond high school level, 63.67% had a partner, and the mean BMI was 29.92 ± 6.89 kg/m^2^. In the diverse quartile of serum manganese levels (Q1–Q4), age, gender, race, educational attainment, marital status, smoking habit, alcohol use, physical activity, BMI, and 24-h total energy intake were significantly different (*p* < 0.05). Compared with the other quartiles, participants in the Q1 group were more likely to be old, male, people who consume more energy in 24 h, and had lower serum klotho levels. Participants in the Q1 group had the highest proportions of smokers, alcohol consumers, and hypertension. Notably, participants with higher manganese quartiles had higher serum klotho levels (Q1: 808.54 ± 256.39 pg/mL; Q2: 854.56 ± 266.13 pg/mL; Q3: 865.13 ± 300.60 pg/mL; and Q4: 871.72 ± 338.85, *p* < 0.001) and lower age (Q1: 58.26 ± 10.56; Q2: 57.34 ± 10.30; Q3: 56.54 ± 10.37; and Q4: 55.17 ± 10.53, *p* < 0.001).

**TABLE 1:** Baseline characteristics of the study participants (n = 3014) recruited from NHANES 2011–2016.

Variable	Serum manganese concentration, μg/L					*p-*value
	Overall	Quartile 1	Quartile 2	Quartile 3	Quartile 4	
		(≤7.27)	(7.27–9.10)	(9.10–11.55)	(>11.55)	
N*	3014	751	754	755	754	
Age, %	56.83 ± 10.50	58.26 ± 10.56	57.34 ± 10.30	56.54 ± 10.37	55.17 ± 10.53	<0.001
Gender, %						<0.001
Male	49.87	60.11	55.29	43.57	34.62	
Female	50.13	39.89	44.71	56.43	65.38	
Race, %						<0.001
Non-Hispanic white	38.79	76.02	76.39	72.02	63.61	
Non-Hispanic black	22.53	13.24	8.90	8.28	6.05	
Mexican American	13.70	4.25	4.73	5.86	11.36	
Other	24.98	6.49	9.99	13.84	18.98	
Educational attainment, %						0.018
Less than high school	23.16	10.84	13.59	14.26	16.83	
High school	21.83	23.47	22.02	19.11	18.91	
College or higher	55.01	65.69	64.40	66.64	64.26	
Marital status, %						0.012
Have a partner	63.67	69.65	73.82	68.33	65.94	
No partner	26.48	22.90	18.65	21.34	25.28	
Unmarried	9.85	7.45	7.53	10.33	8.79	
PIR	2.64 ± 1.65	3.32 ± 1.60	3.34 ± 1.58	3.23 ± 1.58	3.13 ± 1.68	0.054
Smoking habit, %						0.001
Yes	45.62	52.37	47.34	42.52	45.45	
No	54.38	47.63	52.66	57.48	54.55	
Alcohol use, %						<0.001
Yes	72.33	86.27	84.86	77.01	72.49	
No	27.67	13.73	15.14	23.00	27.51	
Diabetes, %						0.121
Yes	18.81	14.70	11.35	14.92	14.67	
No	81.19	85.30	88.65	85.08	85.33	
Hypertension, %						0.056
Yes	45.55	42.79	36.86	42.53	39.81	
No	54.45	57.21	63.14	57.47	60.19	
Physical activity, %						0.035
Vigorous	17.98	21.16	24.84	18.31	19.08	
Moderate	23.89	25.77	24.67	24.56	25.16	
Never	58.13	53.07	50.49	57.13	55.76	
BMI, kg/m^2^	29.92 ± 6.89	29.33 ± 6.37	29.08 ± 6.00	30.73 ± 6.61	30.41 ± 7.18	<0.001
24-h total energy intake, kcal	2030.34 ±	2216.48 ± 894.19	2190.13 ± 915.31	2025.17 ± 805.35	1970.57 ± 790.71	<0.001
	877.34					
klotho (pg/mL)	860.80 ± 312.58	808.54 ± 256.39	854.56 ± 266.13	865.13 ± 300.60	871.72 ± 338.85	<0.001

Notes: Data of normal distribution is displayed as the mean ± standard deviation (SD), while data of skewed distribution is presented as the median (interquartile range: IQR). Categorical variables are demonstrated as a percentage (%). The significance of differences between quartiles is indicated by *p*-values.

Abbreviations: PIR, income-to-poverty ratio; BMI: body mass index.

### 3.2 Association between serum manganese and serum klotho levels

Table 2 shows the association between serum manganese and serum klotho in the three models. In the unadjusted model, serum manganese was markedly positively associated with serum klotho (*β* = 7.30, CI: 4.40–10.20, *p* < 0.001). This association was observed even after partial adjustment (*β* = 6.50, CI: 3.50–9.50, *p* < 0.001) and full adjustment (*β* = 6.30, CI: 3.30–9.40, *p* < 0.001). These three models showed a significantly positive association between serum manganese and serum klotho levels after considering manganese levels as a categorical variable. Taking Q1 as a reference, serum klotho levels increased with increasing serum manganese level quartile (*P* for trend <0.001). Moreover, the restricted cubic spline curve Supplementary Figure S1 showed the non-linear association of serum manganese with serum klotho (*P* for non-linearity *<* 0.05). As shown in Supplementary Table S1, this positive association was significant when lg (manganese) was lower than 0.9 (*p* < 0.05), whereas the association was insignificant when lg (manganese) was higher than 0.9 (*p* > 0.05).

**TABLE 2:** Associations between the serum manganese (μg/L) and serum klotho (pg/mL) levels.

	Model 1	Model 2	Model 3
	β (95% CI, *P*)	β (95% CI, *P*)	β (95% CI, *P*)
Manganese	7.30 (4.40, 10.20)	6.50 (3.50, 9.50)	6.30 (3.30, 9.40)
	<0.001	<0.001	<0.001
Manganese (quartiles)			
Q1	Ref	Ref	Ref
Q2	46.01 (17.38, 74.64)	46.11 (17.54, 74.69)	44.85 (16.30, 73.40)
	<0.010	<0.010	<0.010
Q3	56.58 (27.58, 85.59)	53.32 (24.07, 82.57)	50.90 (21.52, 80.28)
	<0.001	<0.001	<0.001
Q4	63.18 (33.04, 93.33)	55.42 (24.34, 86.50)	52.25 (21.11, 83.40)
	<0.001	<0.001	<0.010
*P* for trend	<0.001	<0.001	<0.001

Notes: Model 1: No adjustment for any variables; Model 2: Adjustment for only 3 variables: age, gender, and race; Model 3: Further adjustment for the following variables: educational attainment, marital status, PIR, smoking habit, alcohol use, physical activity; BMI, 24-h total energy intake, diabetes, and hypertension.

Abbreviations: CI, confidence interval.

### 3.3 Stratified subgroup analysis

**FIGURE 2:**
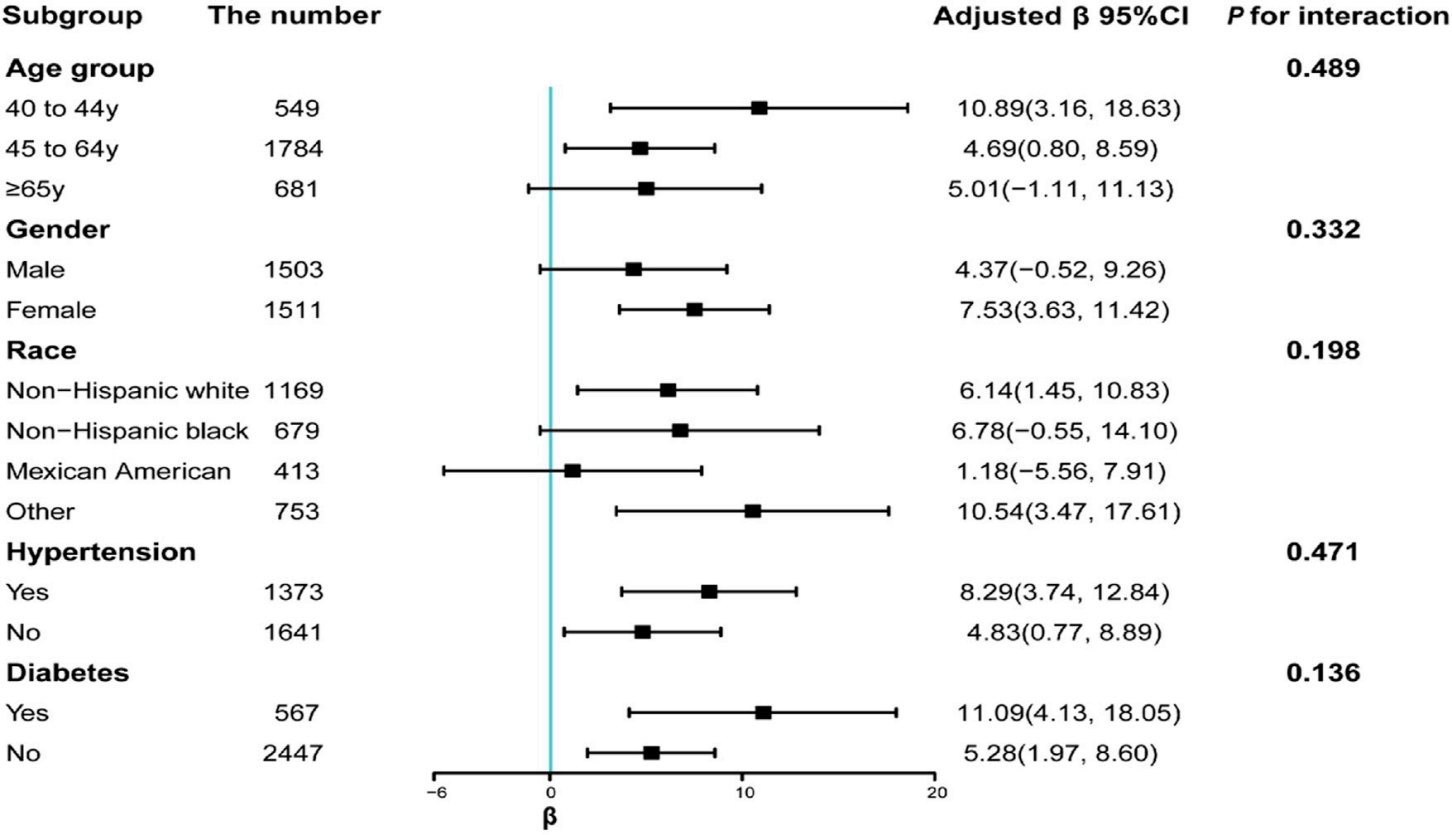
Subgroup analyses of the association between serum manganese and serum klotho levels. Each stratification was adjusted for all factors (i.e., age, gender, race, educational attainment, marital status, PIR, smoking habit, alcohol use, physical activity, BMI, 24-h total energy intake, diabetes, and hypertension), except for the stratification factor itself.

Serum manganese levels were positively associated to serum klotho levels after they were stratified based on variates such as age, gender, race, hypertension, and diabetes (*P* for interaction >0.05) (Figure 2). The following subgroups showed significant positive association between serum manganese levels and serum klotho levels: those aged 40–44 years (*β* = 10.89, CI: 3.16–18.63) or 45–64 years (*β* = 4.69, CI: 0.80–8.59), females (*β* = 7.53, CI: 3.63–11.42), non-Hispanic whites (β = 6.14, CI: 1.45–10.83) or other ethnicities (*β* = 10.54, CI: 3.47–17.61), participants with hypertension (*β* = 8.29, CI: 3.74–12.84) or without hypertension (*β* = 4.83, CI: 0.77–8.89), and participants with diabetes (*β* = 11.09, CI: 4.13–18.05) or without diabetes (*β* = 5.28, CI: 1.97–8.60).

## 4 Discussion

In this study, we observed a non-linear positive association between serum manganese levels and serum klotho levels among middle-aged and elderly in the United States, according to the NHANES conducted from 2011–2016. After adjusting for all covariates, each 1.00 μg/L increase in manganese levels corresponded to the 6.30 pg/mL increase in klotho levels. This significantly positive association was also observed in most subgroups.

The global population is accelerating into the aging stage. According to statistics (Beard and Bloom, 2015), the elderly population will account for 11%–22% by 2050. Therefore, fostering the healthy aging of the elderly is crucial. Nutrients have been found to alleviate aging and age-related diseases among humans. The present study showed a positive association between manganese and the long-lived protein klotho for the first time. As one of the micronutrients, manganese possesses a positive effect of delaying aging (Lv et al., 2021). Lv et al. (2021) found that serum manganese levels found in centenarians (11.41 μg/L) were higher than those found in younger elderly (10.23 μg/L), which indirectly confirmed this result. According to the present study, the positive association between serum manganese and serum klotho levels was significant among the population aged 40–44 years and 45–64 years (*p* < 0.05), whereas the association was non-significant among the population aged 65–80 years (*p* > 0.05). We speculated two reasons to explain this result. First, manganese absorption might differ between older adults and younger adults. The level of the divalent metal transporter-1 was lower in old mice than that in adult mice (Lossow et al., 2020); thus, the old mice inadequately absorbed serum manganese and were prone to manganese poisoning. The second potential reason was the reduction in sample size after grouping. The effect of gender on this association was noteworthy. This positive association was significant among females, whereas it was insignificant among males. We speculated that this result might be caused by the difference in manganese metabolism between women and men. Lee and Kim (Lee and Kim, 2014) discovered that the serum ferritin level was lower in women than in men, which led to the blood manganese levels of women being prone to higher than in men. Lv et al. (2021) also found that the serum manganese levels in males among the elderly were lower than that in females, which might partially explain why women tended to live longer. However, another study failed to show gender-related differences in serum manganese levels among the elderly (Rambousková et al., 2013). These two results are inconsistent. A reasonable speculation is the racial differences between these two studies. The former study included Asian people, whereas the latter included European people. The present study showed that the difference in races among participants could affect the positive association between serum manganese and serum klotho levels. This positive association of serum manganese levels with serum klotho levels was significant in non-Hispanic whites (*p* < 0.05) compared with non-Hispanic black and Mexican Americans (*p* > 0.05).

This study has many advantages: 1) The sample size of this study was large, with a total of 3014 subjects, which was the most representative cross-sectional study on manganese and the longevity protein in Americans. 2) We performed threshold-effect and saturation-effect analyses and determined the lg (manganese) value of 0.90 as the threshold, which has the guiding significance for facilitating the healthy aging of middle-aged and elderly people (Supplementary Table S1). 3) We performed stratification and interaction tests to evaluate the stability of this result further. However, the present study has the following limitations. 1) The data from the questionnaire survey about smoking habit, alcohol use, total energy intake, and physical activity inevitably had some recall bias. 2) Due to the cross-sectional nature of this study, a causal association between blood manganese and blood klotho levels could not be established. 3) Although we adjusted for most confounding factors, a few confounding factors might have been missed, which would have affected the final results. Thus, further prospective studies and basic mechanistic research are crucial to determine the precise effect of manganese levels on klotho levels.

In conclusion, the present study showed a significantly positive association between serum manganese and serum klotho levels after full adjustment for potential confounders. Due to its cross-sectional nature, more basic studies should be performed to clarify the direction and intensity of the effect of manganese on klotho.

## Data Availability

Publicly available datasets were analyzed in this study. This data can be found here: https://wwwn.cdc.gov/nchs/nhanes/Default.aspx
