# Supplementary material for: Association between serum manganese and serum klotho in a 40–80-year-old American population from NHANES 2011–2016
Source: Front Aging. 2023 Mar 8;4:1120823. doi: 10.3389/fragi.2023.1120823 (PMC10031017; doi:10.3389/fragi.2023.1120823)
Supplement: Supplementary file 1 [file Table1.DOCX]

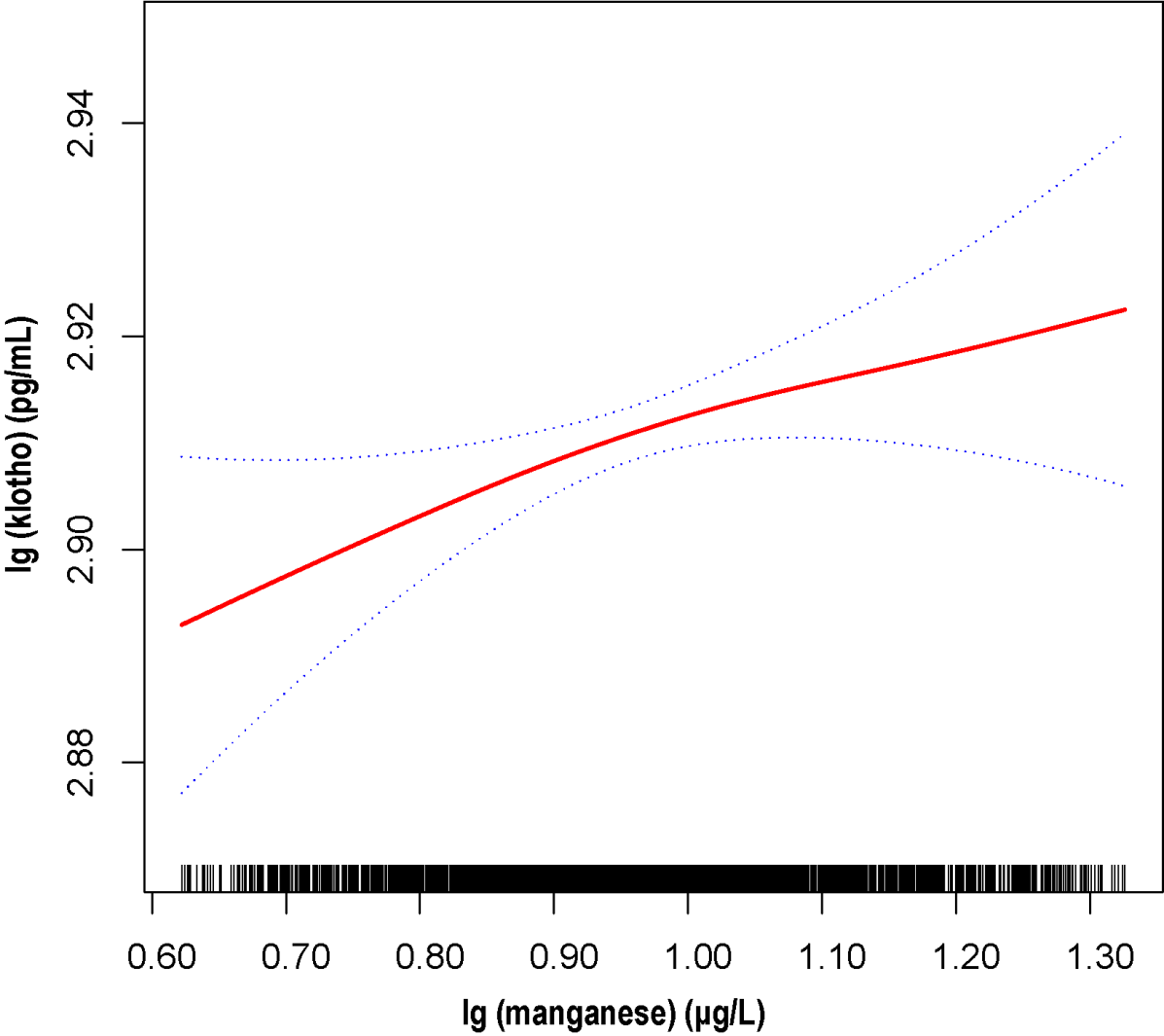


**Supplementary Figure 1.** The restricted cubic spline curve of correlation between the serum manganese and serum klotho levels.

Note: Manganese and klotho levels were lg transformed. A nonlinear association between them was detected after adjusting for all covariates.

**Supplementary Table 1.** Saturation effect analysis before and after adjusting for the effect modifier.

|  | | Before adjustment | After adjustment |
| --- | --- | --- | --- |
| Mode Ⅰ | β value | 0.10 (0.0, 0.1) <0.01 | 0.10 (0.0, 0.1) <0.01 |
| Mode Ⅱ | Breakpoint (K) | 0.90 | 0.90 |
|  | β1 (<0.9) | 0.20(0.10, 0.20) <0.01 | 0.10 (0.10, 0.20) <0.01 |
|  | β2 (>0.9) | 0.00(0.00, 0.10) 0.30 | 0.00(0.00, 0.10) 0.61 |
|  | β2/β1 | -0.10(-0.20, 0.00) 0.04 | -0.10(-0.30, 0.00) 0.02 |
|  | Logarithmic likelihood ratio test *P* value | 0.04 | 0.02 |

Note: Adjustment for all modifiers: age, gender, race, educational attainment, marital status, PIR, smoking habit, alcohol use, diabetes, hypertension, physical activity, BMI, and 24-h total energy intake.
