# Supplementary material for: Association between serum manganese and serum klotho in a 40–80-year-old American population from NHANES 2011–2016
Source: Front Aging. 2023 Mar 8;4:1120823. doi: 10.3389/fragi.2023.1120823 (PMC10031017; doi:10.3389/fragi.2023.1120823)
Supplement: Supplementary file 2 [file Table2.DOCX]

**Table 1**. Baseline characteristics of the study participants (n = 3014) [recruited](https://cn.bing.com/dict/search?q=recruited&FORM=BDVSP6&cc=cn) from NHANES 2011–2016.

| Variable | Serum manganese concentration, μg/L | | | | | *P-*value |
| --- | --- | --- | --- | --- | --- | --- |
|  | Overall | Quartile 1 | Quartile 2 | Quartile 3 | Quartile 4 |  |
|  |  | (≤7.27) | (7.27–9.10) | (9.10–11.55) | (>11.55) |  |
| N* | 3014 | 751 | 754 | 755 | 754 |  |
| Age, % | 56.83 ±10.50 | 58.26 ±10.56 | 57.34 ±10.30 | 56.54 ±10.37 | 55.17 ±10.53 | <0.001 |
| Gender, % |  |  |  |  |  | <0.001 |
| Male | 49.87 | 60.11 | 55.29 | 43.57 | 34.62 |  |
| Female | 50.13 | 39.89 | 44.71 | 56.43 | 65.38 |  |
| Race, % |  |  |  |  |  | <0.001 |
| Non-Hispanic white | 38.79 | 76.02 | 76.39 | 72.02 | 63.61 |  |
| Non-Hispanic black | 22.53 | 13.24 | 8.90 | 8.28 | 6.05 |  |
| Mexican American | 13.70 | 4.25 | 4.73 | 5.86 | 11.36 |  |
| Other | 24.98 | 6.49 | 9.99 | 13.84 | 18.98 |  |
| Educational attainment, % |  |  |  |  |  | 0.018 |
| Less than high school | 23.16 | 10.84 | 13.59 | 14.26 | 16.83 |  |
| High school | 21.83 | 23.47 | 22.02 | 19.11 | 18.91 |  |
| College or higher | 55.01 | 65.69 | 64.40 | 66.64 | 64.26 |  |
| Marital status, % |  |  |  |  |  | 0.012 |
| Have a partner | 63.67 | 69.65 | 73.82 | 68.33 | 65.94 |  |
| No partner | 26.48 | 22.90 | 18.65 | 21.34 | 25.28 |  |
| Unmarried | 9.85 | 7.45 | 7.53 | 10.33 | 8.79 |  |
| PIR | 2.64 ±1.65 | 3.32 ±1.60 | 3.34 ±1.58 | 3.23 ±1.58 | 3.13 ±1.68 | 0.054 |
| Smoking habit, % |  |  |  |  |  | 0.001 |
| Yes | 45.62 | 52.37 | 47.34 | 42.52 | 45.45 |  |
| No | 54.38 | 47.63 | 52.66 | 57.48 | 54.55 |  |
| Alcohol use, % |  |  |  |  |  | <0.001 |
| Yes | 72.33 | 86.27 | 84.86 | 77.01 | 72.49 |  |
| No | 27.67 | 13.73 | 15.14 | 23.00 | 27.51 |  |
| Diabetes, % |  |  |  |  |  | 0.121 |
| Yes | 18.81 | 14.70 | 11.35 | 14.92 | 14.67 |  |
| No | 81.19 | 85.30 | 88.65 | 85.08 | 85.33 |  |
| Hypertension, % |  |  |  |  |  | 0.056 |
| Yes | 45.55 | 42.79 | 36.86 | 42.53 | 39.81 |  |
| No | 54.45 | 57.21 | 63.14 | 57.47 | 60.19 |  |
| Physical activity, % |  |  |  |  |  | 0.035 |
| Vigorous | 17.98 | 21.16 | 24.84 | 18.31 | 19.08 |  |
| Moderate | 23.89 | 25.77 | 24.67 | 24.56 | 25.16 |  |
| Never | 58.13 | 53.07 | 50.49 | 57.13 | 55.76 |  |
| BMI, kg/m^2^ | 29.92 ±6.89 | 29.33 ±6.37 | 29.08 ±6.00 | 30.73 ±6.61 | 30.41 ±7.18 | <0.001 |
| 24-h total energy intake, kcal | 2030.34 ±  877.34 | 2216.48 ± 894.19 | 2190.13 ± 915.31 | 2025.17 ± 805.35 | 1970.57 ± 790.71 | <0.001 |
| klotho (pg/mL) | 860.80 ±312.58 | 808.54 ±256.39 | 854.56 ±266.13 | 865.13 ±300.60 | 871.72 ±338.85 | <0.001 |

Notes: Data of normal distribution is displayed as the mean ±standard deviation (SD), while data of skewed distribution is presented as the median (interquartile range: IQR). Categorical variables are demonstrated as a percentage (%). The significance of differences between quartiles is indicated by *P*-values.

Abbreviations: PIR: income-to-poverty ratio; BMI: body mass index

**Table 2.** Associations between the serum manganese (μg/L) and serum klotho (pg/mL) levels.

|  | Model 1 | Model 2 | Model 3 |
| --- | --- | --- | --- |
|  | β (95% CI, *P*) | β (95% CI, *P*) | β (95% CI, *P*) |
| Manganese | 7.30 (4.40, 10.20) | 6.50 (3.50, 9.50) | 6.30 (3.30, 9.40) |
|  | <0.001 | <0.001 | <0.001 |
| Manganese (quartiles) |  |  |  |
| Q1 | Ref | Ref | Ref |
| Q2 | 46.01 (17.38, 74.64) | 46.11 (17.54, 74.69) | 44.85 (16.30, 73.40) |
|  | < 0.010 | < 0.010 | < 0.010 |
| Q3 | 56.58 (27.58, 85.59) | 53.32 (24.07, 82.57) | 50.90 (21.52, 80.28) |
|  | < 0.001 | < 0.001 | < 0.001 |
| Q4 | 63.18 (33.04, 93.33) | 55.42(24.34, 86.50) | 52.25 (21.11, 83.40) |
|  | < 0.001 | < 0.001 | < 0.010 |
| *P* for trend | <0.001 | <0.001 | <0.001 |

Notes: Model 1: No adjustment for any variables; Model 2: Adjustment for only 3 variables: age, gender, and race; Model 3: Further adjustment for the following variables: educational attainment, marital status, PIR, smoking habit, alcohol use, physical activity, BMI, 24-h total energy intake, diabetes, and hypertension.

Abbreviations: CI: confidence interval
